# Fatigue state and external load effects on firefighter sprinting biomechanics

**DOI:** 10.3389/fbioe.2025.1677575

**Published:** 2025-12-05

**Authors:** Chuangui Mao, Ziwen Wang, Xinxin Zhang, Xiaoyi Ma, Sihang Zeng, Yunfei Hao, Weiguo Liu, Yu Miao

**Affiliations:** 1 College of Physical Education and Health, Guangxi Normal University, Guilin, China; 2 School of Physical Education, Shaanxi Normal University, Xi’an, China

**Keywords:** Sprinting, load carriage, fatigue, firefighter, Biomechanics

## Abstract

**Objective:**

This study investigates the biomechanical effects of fatigue and load on professional firefighters during sprinting, aiming to elucidate movement adaptations and injury risks under occupational rescue scenarios. We hypothesized that fatigue and load would independently impair sprinting performance and alter lower-limb biomechanics, leading to compensatory increases in joint moments and muscle activation.

**Methods:**

Sixteen firefighters (23.13 ± 3.52 years) performed 20-m sprints carrying different loads (10–30 kg) in pre-/post-fatigue states. The statistical analysis was performed using SPSS and SPM1d for the two-way repeated-measures ANOVA.

**Results:**

There were significant differences in the gait speed (*F =* 14.019, *p <* 0.001, *η* ^2^ = 0.683), step length (*F =* 30.512, *p <* 0.001, *η* ^2^ = 0.685), stance time (*F =* 20.256, *p <* 0.001, *η* ^2^ = 0.591), rectus abdominis (*F =* 6.757, *p =* 0.004, *η* ^2^ = 0.326), and rectus femoris (*F =* 13.434, *p =* 0.002, *η* ^2^ = 0.490) under different load tasks. The results of SPM1d revealed significant differences in the hip flexion/extension angles (*F =* 5.626, *p =* 0.049, 89.38%–99.30%), hip flexion/extension moments (*F =* 9.981, *p <* 0.001, 92.16%–100%), and ankle dorsiflexion/plantar flexion moments (*F =* 8.852, *p =* 0.003, 66.19%–78.98%) during the stance phase.

**Conclusion:**

When sprinting with external weight constraints, gait metrics are negatively impacted, and compensating kinetic strategies increase push-off power from the hip and ankle of the dominant leg. These adaptations are further reflected in increased activation of the muscles around the hip joint of the firefighters.

## 1 Introduction

According to the latest statistics from the National Fire and Rescue Administration, China’s fire and rescue teams responded to 2.053 million cases (January-October 2024), up 11% from 1.843 million cases in the same period in 2023 (National Fire and Rescue Administration, 2025), indicating that fire and rescue teams handle nearly five incidents per minute. Beyond fire prevention and life rescue, firefighters perform disaster relief and traffic accident missions. Fatigue and overexertion are the most often reported causes of injury (Aurora et al., 2020), with over 165 firefighters losing their lives and 1,300 more suffering serious injuries in the past 5 years (Liu, 2023). Consequently, fatigue-induced movement degradation is a primary causal pathway in occupational injuries, necessitating a mechanistic investigation of fatigued rescue capabilities.

The safety of firefighters’ work has been improved by equipment developed with protective and biomechanical considerations in modern design. For example, wearing firefighting suits has been shown to impair dynamic stability, joint range of motion, and gait kinematics (such as step length and gait speed) (Liu et al., 2022). Additionally, firefighting boots would increase the risk of injury by decreasing ankle flexibility, increasing lower limb stress, and placing additional loading on the lumbar spine (Vu et al., 2017; Tian et al., 2017). These studies reflect improvements in firefighting gear, exploring how equipment changes human movement patterns. And in the past 2 years, researchers found that the Self-Contained Breathing Apparatus raises the pressures of the knee joint during running, and they investigated its effect on muscle fatigue in the shoulders, back, and legs of firefighters (Wang et al., 2023; Huimin et al., 2024). The above studies analyze the main factors affecting fatigue in firefighters at work and identify control measures to mitigate the risks associated with such fatigue (Marvin et al., 2023; Schmitz et al., 2025). The previous research has proved that fatigue poses a major risk to firefighters’ health and safety, and the consequences include a higher chance of accidents and a decline in cognitive ability (Schmitz et al., 2025; Bustos et al., 2022). Besides, a recent review indicates that load carriage is a leading mechanism for injury among soldiers (Robin et al., 2025a). However, few studies have been done on how fatigue and load carriage impact a firefighter’s capacity to rescue. The combined effects of fatigue and dynamic rescue maneuvers especially lack quantitative biomechanical characterization. Therefore, investigating the effects of fatigue and load is essential to improving their work efficiency and occupational quality of life in rescue situations and lowering their health risks and injuries.

This study investigates the biomechanical characteristics of professional firefighters during loaded sprinting under pre-fatigue and post-fatigue states. Based on previous studies, we hypothesize that fatigue and load magnitude dominantly affect gait parameters and biomechanical measurement parameters related to the hip joint. Furthermore, employing one-dimensional Statistical Parametric Mapping (SPM1d), we analyze continuous movement phases, overcoming limitations of traditional methods that assess discrete time points (Yona et al., 2024; Zijun et al., 2024; Mao et al., 2023). As a statistical method based on random field theory, it conducts hypothesis testing and significance judgments on the entire curve rather than at specific time points. It offers a novel methodological framework for clarifying the biomechanical mechanisms of occupational injuries by revealing subtle differences with greater precision, comprehensiveness, and visual clarity for continuous data and other metrics in this experiment. It will help identify potential mechanisms and influencing factors behind rescue movement adaptations.

## 2 Materials and methods

### 2.1 Participants

Sixteen professional firefighters (age: 23.13 ± 3.52 years; height: 170.81 ± 2.67 cm; weight: 64.35 ± 4.83 kg; years of service: 4.25 ± 2.11 years) from the local fire department in Guilin, Guangxi Province, were recruited. The effectiveness of the sample size was calculated in G*Power software (ver. 3.1.9.7; Heinrich-Heine-Universität Düsseldorf, Düsseldorf, Germany). *A priori* power analysis was conducted using G*Power to determine the minimum sample size required to detect the effect. Based on an effect size of η^2^ = 0.26 observed in a similar study on the maximum knee flexion moment (Xinxin et al., 2024), and with α set at 0.05 and power (1-β) at 0.95 for a repeated-measures ANOVA with within-subjects factors, the analysis indicated that a minimum sample size of 6 participants was required. Before our experiment, all participants could maintain a training intensity of at least 6 times per week and met the Chinese firefighters’ physical fitness assessment standards. All participants exhibited a rearfoot strike pattern during running. The dominant leg—defined as the preferred leg for kicking a ball based on self-reported habit—was identified for each participant, and all biomechanical analyses were performed on this limb to ensure data independence (Xinxin et al., 2024). Exclusion criteria included: history of serious injuries or prolonged medication use during the previous 12 months. All participants were fully informed about the experimental content and procedures, provided written informed consent, and obtained ethical approval from the Guangxi Normal University Ethics Committee (Approval No.: 20230419001).

### 2.2 Fatigue protocol

**FIGURE 1:**
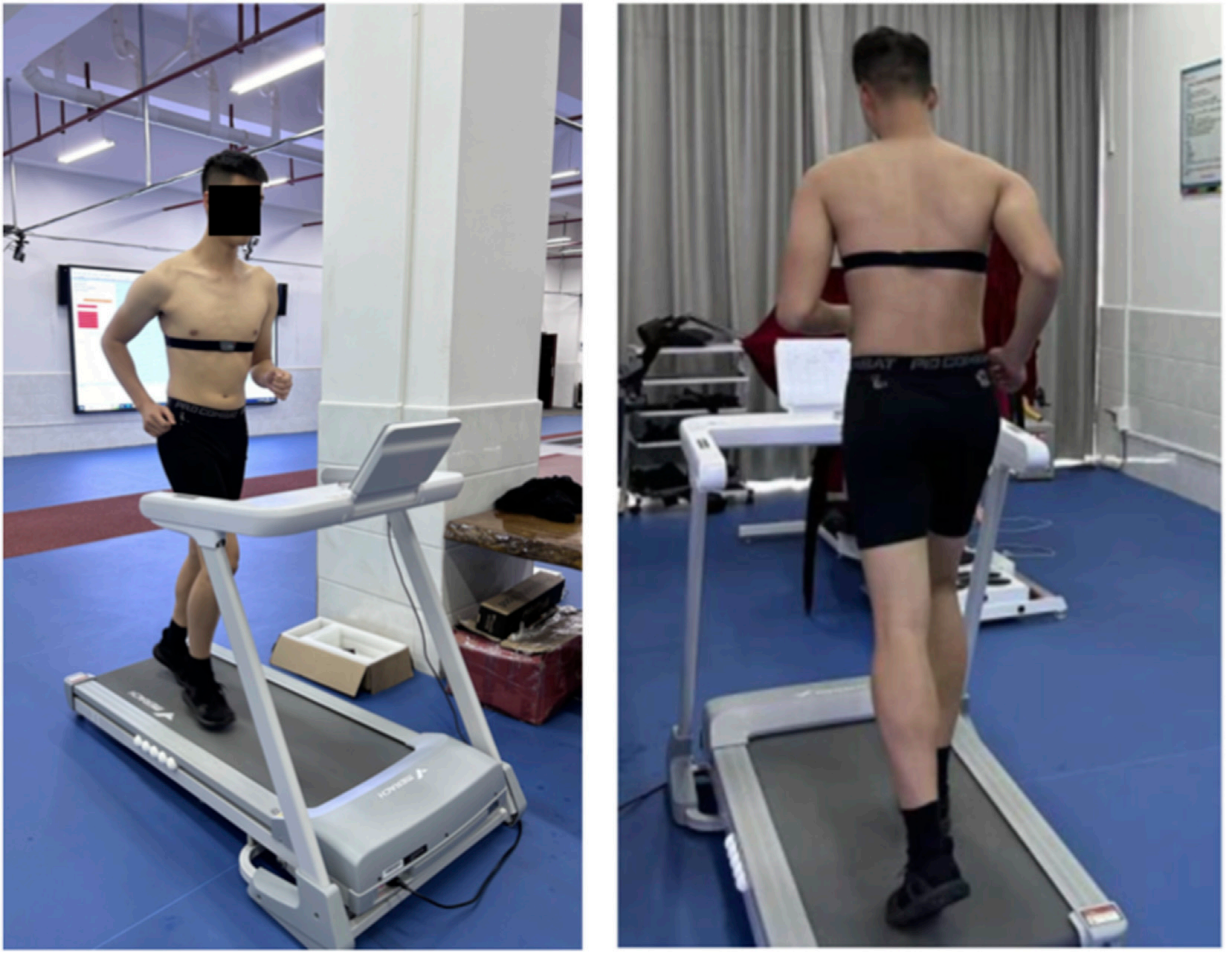
Execution of the standardized fatigue protocol.

Participants wore uniform pants and shoes during a standardized 10-min warm-up, which included 5 min of jogging and 5 min of lower limb dynamic stretching, before testing. After warming up, firefighters wore heart rate monitors (Polar H9, Polar Electro Oy, Finland) and completed a whole-body fatigue protocol using a maximal graded treadmill test (Figure 1) (Steib et al., 2013; Brett et al., 2010). The fatigue protocol using a treadmill proceeded as follows: Firefighters began treadmill testing at an initial speed of 5 km/h with 0% slope after warm-up. While maintaining a constant speed, the slope increased by 2% every 2 min, reaching 14%. The slope remained fixed while speed increased by 0.5 km/h per minute. Participants were instructed to run as long as possible until complete exhaustion, and verbal encouragement was provided throughout the test, particularly toward the point of volitional exhaustion. Heart rate was monitored using a Polar heart rate monitor along with perceived fatigue severity judged by the Borg 6–20 rate of perceived exertion (RPE) scale. Fatigue was defined as achieving 90% of the age-predicted maximum heart rate (220-age) combined with an inability to maintain the required speed, or participants reported their fatigue to be severe (20 on the Borg scale); the test was then terminated (Nelson et al., 2012).

### 2.3 Experiment design

**FIGURE 2:**
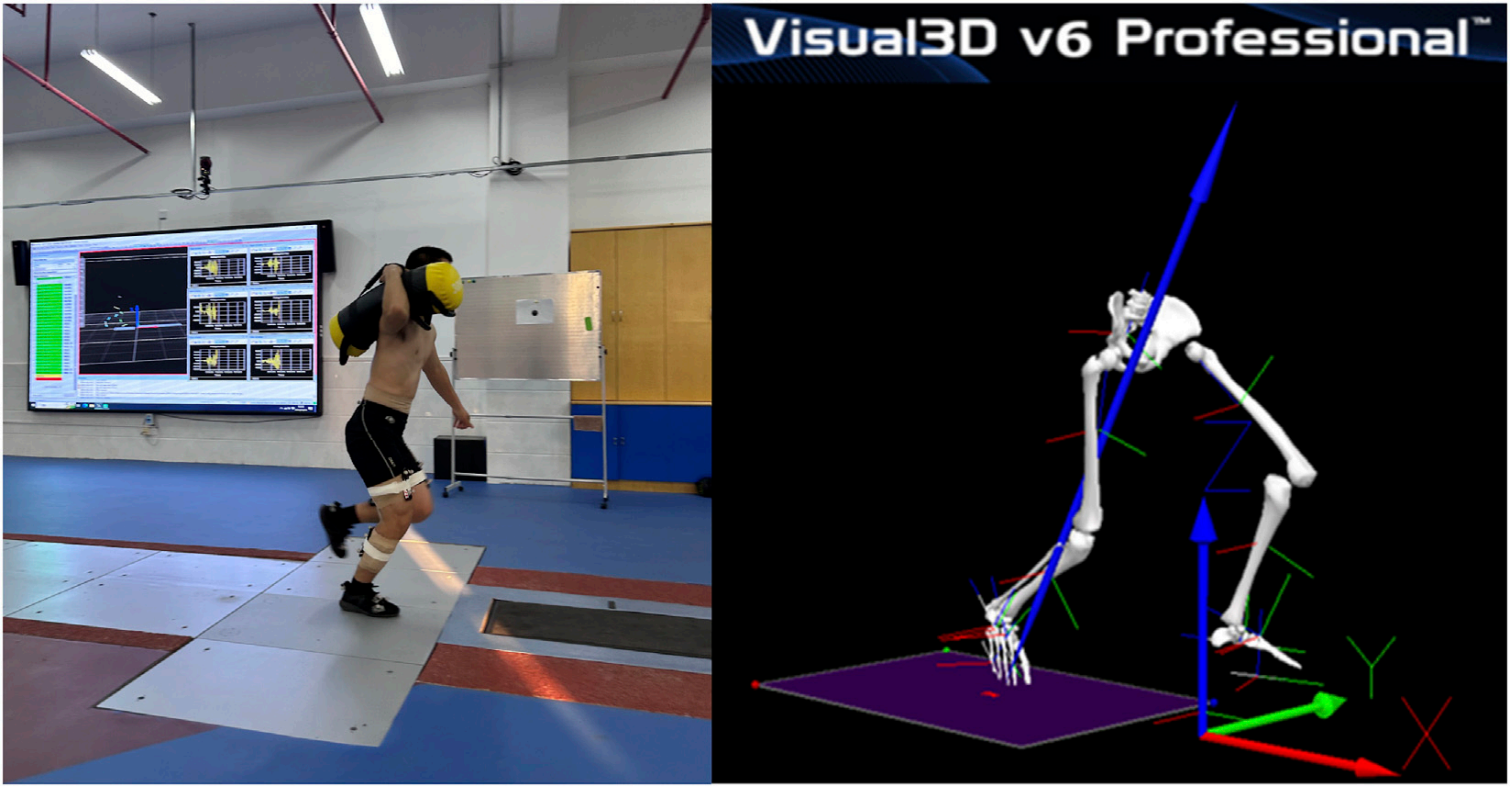
Field execution and biomechanical modeling of loaded sprinting.

Participants completed two testing sessions in randomized order: pre-fatigue and post-fatigue. Kinematic data were captured using eight infrared cameras (Oqus 600, Qualisys AB, Sweden) at 200 Hz, with 36 retroreflective markers (14 mm diameter) placed on lower limb landmarks according to the Helen Hayes marker set. Kinetic data were synchronously collected via a force plate (Type 9287CA, Kistler Group, Switzerland) at 1,000 Hz. Surface electromyography (EMG) signals were recorded using an Ultium EMG sensor system (Noraxon USA Inc., USA) at 2000 Hz. The preparation of all devices must be completed within 10 min after the fatigue protocol to prevent the dissipation of the fatigue effect. Static trials were acquired for skeletal modeling and subsequent processing. During dynamic data collection, participants performed maximal-effort sprint trials along a straight runway to standardize starting positions. In the formal experiment, firefighters sprinted 20 m under three load conditions (10 kg, 20 kg, and 30 kg carried on the dominant shoulder) (Tang et al., 2025)—a loading pattern representative of rescue tasks commonly used in training (Xinxin et al., 2024). The order of load conditions within the post-fatigue session was randomized. Each participant performed three valid trials per load condition, where a trial was considered valid only if the entire dominant foot naturally contacted the force plate without marker separation (Figure 2). A rest period of at least 2 min was provided between consecutive loaded sprinting trials. All sixteen participants completed all trials under both pre-fatigue and post-fatigue conditions. No data were excluded due to technical errors or participant dropout, resulting in a complete dataset for all dependent measures.

### 2.4 Data processing

Kinematic and kinetic data were synchronized and processed in Visual 3D software. A fourth-order Butterworth low-pass filter was applied at 14 Hz for kinematics and 50 Hz for kinetics (Mao et al., 2023). Vertical ground reaction force (vGRF) thresholds (10 N) were identified to determine initial contact and toe-off events, thereby defining the stance phase (Chuangui et al., 2024). Time-normalized data (101 interpolated points per stance phase) were used for SPM1d analysis. For SPM1d analysis, lower limb joint angles, joint moments, vertical ground reaction force (vGRF), and sagittal-plane center-of-mass to center-of-pressure (COM-COP) inclination angles were analyzed alongside, which were calculated using established methods (Zijun et al., 2024; Lin et al., 2021). Joint moments were normalized to body weight multiplied by height (BW × HT), and vGRF was normalized by body weight. Based on prior studies, gait parameters (gait speed, step length, step width, stance time, flight time, peak impact force, and peak propulsive force) were selected to characterize firefighters’ biomechanical profiles during sprinting (John et al., 2008; Pedro et al., 2008; Matt et al., 2014). This research analyzed the integral electromyographic (iEMG) of rectus femoris, biceps femoris, gastrocnemius lateralis, tibialis anterior, and gluteus maximus during the stance phase. After being filtered, the iEMG data during the test were normalized by dividing by the MVC data, which was extracted from the middle 3s of a 5s period (kept stable for 5 s) for averaging (Zijun et al., 2024).

### 2.5 Statistical analysis

Statistical analyses were performed using SPSS (SPSS Statistics v 25, IBM Corp., U.S.) and Matlab (MATLAB 2016a; MathWorks Inc., U.S.). Using two-way repeated-measures ANOVA for discrete variables (spatiotemporal parameters) in SPSS and for the continuous data via SPM1d in MATLAB to examine the effects of fatigue (pre/post) and load (10 kg, 20 kg, 30 kg) on sprint biomechanics. Normality was assessed using Shapiro-Wilk tests to compare values. If a significant interaction or main effect was found, the Bonferroni *post hoc* test was used for multiple comparisons. Statistical significance was set at α = 0.05. Based on previous studies, partial eta-squared ( $\eta_{p}^{2}$ ) was used to report effect sizes, specifying 0.1–0.25 as a small effect size, 0.25–0.4 as a medium effect size, and greater than 0.4 as a large effect size.

## 3 Results

The fatigue protocol lasted on average 13.5 ± 2.1 min. Mean heart rate of participants increased significantly from 63.4 ± 4.2 bpm at rest to 186.2 ± 5.8 bpm at exhaustion, reaching 95% of the maximum HR (%HRmax).

The result showed no significant fatigue or interaction effects (Table 1). Significant load effects were observed in: gait speed (*F(2,30)* = 14.019, *p <* 0.001, *η* ^2^ = 0.683), step width (*F(2,30)* = 6.170, *p =* 0.006, *η* ^2^ = 0.306), step length (*F(2,30)* = 30.512, *p <* 0.001, *η* ^2^ = 0.685), stance time (*F(2,30)* = 20.256, *p <* 0.001, *η* ^2^ = 0.591), flight time (*F(2,30)* = 12.545, *p <* 0.001, *η* ^2^ = 0.659), peak impact force (*F(2,30)* = 11.824, *p <* 0.001, *η* ^2^ = 0.458), peak propulsive force (*F(2,30)* = 9.888, *p <* 0.001, *η* ^2^ = 0.414).

**TABLE 1:** Comparison of gait parameters during loaded sprinting (N = 16).

Variable	State	10 kg	20 kg	30 kg	*p*-values		
					Fatigue	Load	Interaction
Gait speed (m/s)	Pre-F	4.66 ± 0.40^a^	4.36 ± 0.37^bb^	4.10 ± 0.34^cc^	0.446	*<*0.001^**^	0.518
	Post-F	4.41 ± 0.40	4.24 ± 0.36^b^	4.06 ± 0.39^c^			
Step width (m)	Pre-F	0.11 ± 0.05	0.11 ± 0.03	0.09 ± 0.04	0.210	0.006^**^	0.233
	Post-F	0.08 ± 0.04^a^	0.10 ± 0.03^b^	0.06 ± 0.03			
Step length (m)	Pre-F	1.49 ± 0.22^a^	1.39 ± 0.18^bb^	1.29 ± 0.14^cc^	0.585	*<*0.001^**^	0.819
	Post-F	1.53 ± 0.24	1.45 ± 0.17^bb^	1.35 ± 0.15^cc^			
Stance time (s)	Pre-F	0.21 ± 0.02	0.23 ± 0.03^bb^	0.25 ± 0.03^cc^	0.708	*<*0.001^**^	0.396
	Post-F	0.22 ± 0.02^a^	0.24 ± 0.02	0.24 ± 0.04^c^			
Flight time (s)	Pre-F	0.11 ± 0.03	0.09 ± 0.03^bb^	0.07 ± 0.03^cc^	0.255	*<*0.001^**^	0.483
	Post-F	0.13 ± 0.05^a^	0.11 ± 0.03	0.09 ± 0.03^c^			
Peak impact force (%BW)	Pre-F	2.17 ± 0.64	2.35 ± 0.64^b^	2.58 ± 0.47^c^	0.820	*<*0.001^**^	0.386
	Post-F	2.10 ± 0.38^a^	2.41 ± 0.28	2.44 ± 0.37			
Peak propulsive force (%BW)	Pre-F	2.53 ± 0.25^a^	2.64 ± 0.33	2.67 ± 0.23^c^	0.491	*<*0.001^**^	0.373
	Post-F	2.48 ± 0.23	2.51 ± 0.23	2.61 ± 0.25^c^			

The statistical significance is marked with *. a: significant difference (*p* < 0.05) between 10 kg and 20 kg. b: significant difference (*p* < 0.05) between 20 kg and 30 kg. c: significant difference (*p* < 0.05) between 10 kg and 30 kg. The double letter indicates *p* < 0.01, respectively. Pre-F, pre-fatigue; Post-F, post-fatigue.

The results showed no significant differences in fatigue or interaction effects (Table 2). Significant load effects were observed in: rectus abdominis (*F(2,30)* = 6.757, *p =* 0.004, *η* ^2^ = 0.326), rectus femoris (*F(2,30)* = 13.434, *p =* 0.002, *η* ^2^ = 0.490), biceps femoris (*F(2,30)* = 5.011, *p =* 0.014, *η* ^2^ = 0.264).

**TABLE 2:** Comparison of iEMG during the stance phase (N = 16).

Variable	State	10 kg	20 kg	30 kg	*p*-values		
					Fatigue	Load	Interaction
Gluteus maximus (%)	Pre-F	0.10 ± 0.02	0.10 ± 0.02	0.12 ± 0.02	0.759	0.094	0.319
	Post-F	0.09 ± 0.01	0.10 ± 0.01	0.10 ± 0.01			
Rectus abdominis (%)	Pre-F	0.16 ± 0.02	0.17 ± 0.03	0.18 ± 0.03^c^	0.748	0.004^**^	0.768
	Post-F	0.18 ± 0.02	0.19 ± 0.02	0.20 ± 0.03			
Rectus femoris (%)	Pre-F	0.07 ± 0.01	0.09 ± 0.01^b^	0.12 ± 0.01^cc^	0.148	0.002^**^	0.128
	Post-F	0.12 ± 0.02	0.14 ± 0.02	0.15 ± 0.02			
Biceps femoris (%)	Pre-F	0.12 ± 0.02	0.13 ± 0.02	0.13 ± 0.02	0.877	0.014^*^	0.222
	Post-F	0.12 ± 0.01	0.13 ± 0.01	0.15 ± 0.01			
Gastrocnemius lateral (%)	Pre-F	0.29 ± 0.05	0.28 ± 0.05	0.32 ± 0.06	0.570	0.371	0.543
	Post-F	0.23 ± 0.02	0.27 ± 0.03	0.26 ± 0.02			
Tibialis anterior (%)	Pre-F	0.06 ± 0.02	0.04 ± 0.01	0.04 ± 0.01	0.631	0.475	0.433
	Post-F	0.05 ± 0.02	0.08 ± 0.03	0.04 ± 0.01			

The statistical significance is marked with *. a: significant difference (*p* < 0.05) between 10 kg and 20 kg. b: significant difference (*p* < 0.05) between 20 kg and 30 kg. c: significant difference (*p* < 0.05) between 10 kg and 30 kg. The double letter indicates *p* < 0.01, respectively. Pre-F, pre-fatigue; Post-F, post-fatigue.

**FIGURE 3:**
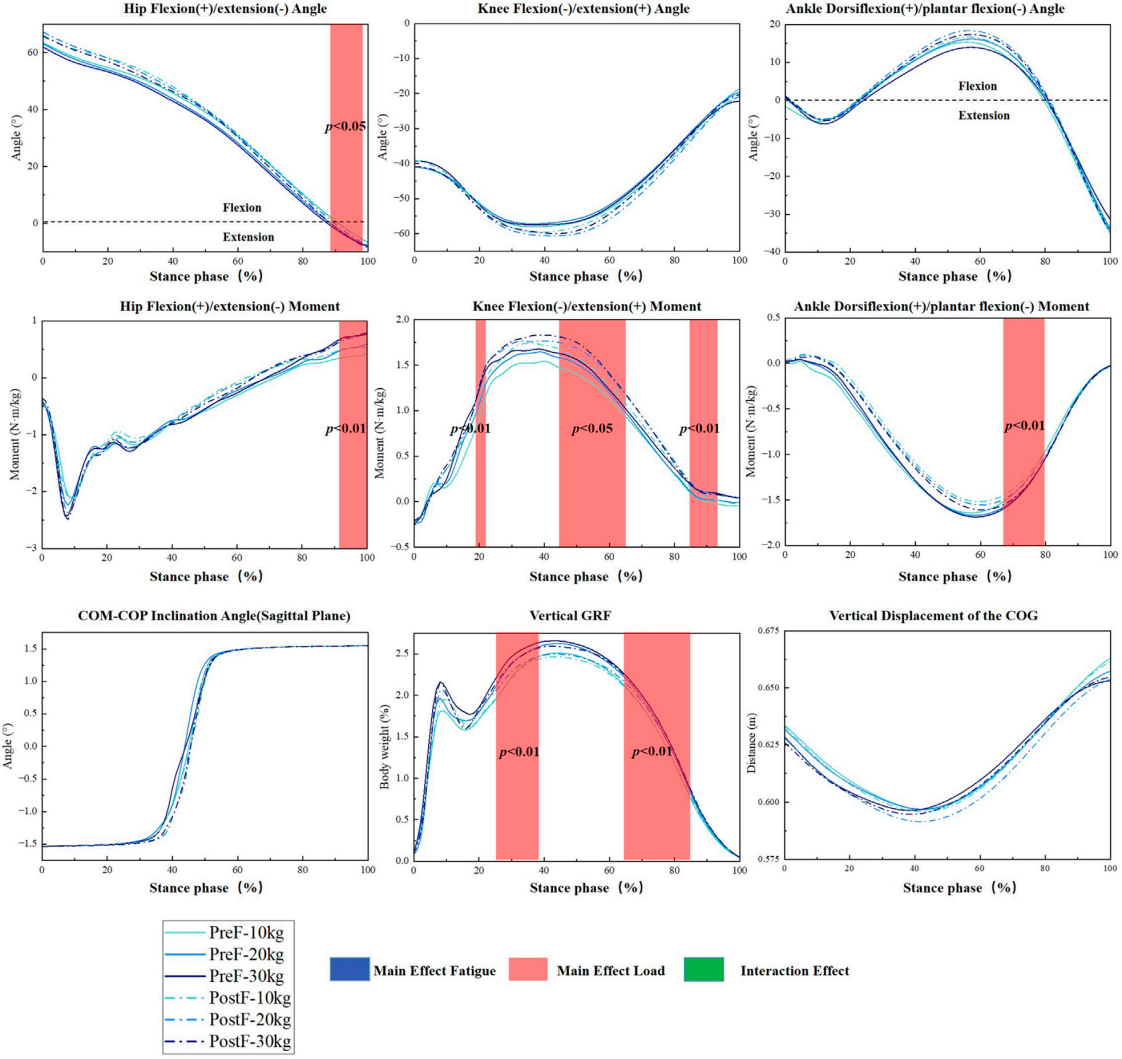
SPM1d analysis of kinematic and kinetic parameters during the stance phase of loaded sprinting. Time-normalized curves under different load conditions (10 kg, 20 kg, 30 kg) in pre-fatigue (solid lines) and post-fatigue (dashed lines) states. The red shaded areas highlight the specific periods within the stance phase where a significant main effect of load was detected (*p* < 0.05).

Figure 3 shows no significant fatigue or interaction effects during the stance phase. Significant differences existed in the dominant leg’s hip flexion/extension angle (*F =* 5.626, *p =* 0.049, 89.38%–99.30%). There were significant differences in the hip flexion/extension moment (*F =* 9.981, *p <* 0.001, 92.16%–100%), knee flexion/extension moment (*F =* 9.760, *p =* 0.046, 19.81%–21.31%; *F =* 9.760, *p <* 0.001, 46.78%–66.40%; *F =* 9.760, *p =* 0.002, 91.20%–100%) and ankle dorsiflexion/plantar flexion moments (*F =* 8.852, *p =* 0.003, 66.19%–78.98%) of the dominant leg. There were significant differences in the vertical GRF (*F =* 10.012, *p <* 0.001, 24.46%–38.84%; *F =* 10.012, *p <* 0.001, 65.93%–84.30%).

## 4 Discussion

Firefighting often requires maximal-effort sprints under heavy load and high fatigue to achieve critical objectives, such as reaching a victim or escaping a hazard. The biomechanical adaptations under these conditions directly determine operational success and injury risk. This study demonstrates biomechanical differences in professional firefighters during loaded sprinting under pre-fatigue and post-fatigue states. (Table 1; Figure 2). Importantly, the dominant influence of load, rather than fatigue, reveals a unique aspect of this specific firefighter cohort.

This attenuated fatigue effect may be attributable to the specific demographic profile of Chinese firefighters. Unlike career-based systems (e.g., U.S., EU), China relies predominantly on a conscripted service model with mandatory short-term enlistment, resulting in a younger cohort (Joel et al., 2025; Andria et al., 2024; Jaron et al., 2023). Younger individuals typically exhibit better recovery kinetics and may be less susceptible to rapid declines in anaerobic performance under fatigue compared to older populations experiencing natural aerobic capacity decline (Clare et al., 2016). While this physiological resilience is an operational asset, allowing these young firefighters to maintain sprint performance under acute fatigue, it also highlights a critical profession-specific consideration. That said, the possibility that the selected biomechanical variables were not sufficiently sensitive to detect subtle neuromuscular changes, or that the fatigue protocol emphasized aerobic/metabolic fatigue over neuromuscular aspects, should also be considered in the interpretation. Future research could employ machine learning algorithms for gait pattern recognition, offering a more sensitive and holistic approach (Xu et al., 2024; Xu et al., 2022). Nevertheless, the findings underscore the notable load-adaptive capabilities of this population, even under acute fatigue, suggesting that such physiological resilience likely contributed to the non-significant fatigue effects observed.

Turning to the dominant load influence, our findings reveal biomechanical adaptations that directly affect firefighter operational efficiency and injury risk. The load-induced reductions in gait speed and step length and increased stance time and peak forces (Table 1) directly translate to slower response times during rescue sprints and elevated mechanical stress on the musculoskeletal system (John et al., 2008; Pedro et al., 2008; Matt et al., 2014). The observed reduction in step width under heavy load (30 kg ≈ 50% body mass) may represent a strategic deviation from the optimal U-shaped relationship for stability and energy expenditure (Xinxin et al., 2024; Christopher and Rodger, 2011; Yuan et al., 2024). In an occupational context, this suggests firefighters may unconsciously prioritize energy conservation over stability when carrying heavy equipment, potentially increasing the risk of trips and falls on uneven emergency terrains.

The load-induced reductions in gait speed and step length align with evidence that running with 19% added body weight increases peak vGRF by 4.7%. Reduced flight time may reflect impaired generation of vertically-directed power, limiting the rise of the center of mass and consequently restricting leg extension and stride length (Pedro et al., 2008; Matt et al., 2014). The previous study showed that peak gluteus maximus force during the early stance phase supports propulsion by facilitating powerful leg extension (Róisín et al., 2017). A systematic review summarized that stance time and flight time are more affected by load than cadence and step length, and the decrease in speed is due to a significant reduction in horizontal force and power during the stance phase of loaded running (Macadam et al., 2022). In addition, the researchers believe that the change in the contact time between the foot and the ground may be a natural protective response of the human body, aiming to maximize stability and reduce injury risk when coping with movement challenges (e.g., disturbed balance or altered gait patterns) (Tian et al., 2017; Sun et al., 2015). Finally, some studies believe an additional load primarily changes vertical impulse rather than horizontal force characteristics (Macadam et al., 2022; Sam et al., 2021). Meanwhile, the gastrocnemius (contributing to ankle plantar-flexion) and rectus femoris (facilitating knee extension and hip flexion) support vertical stabilization and power transfer during ground contact (Robin et al., 2025b), which is consistent with our results of iEMG (Table 2). These changes in gait parameters are not merely simple mechanical results; they also reflect the active control strategies of the nervous system under load. The shortened stride length and increased ground contact time can be interpreted as the neuromuscular system’s “robustness” strategy (Niamh et al., 2021). The nervous system may enhance the stability control under load impact by reducing the duration of the swing phase and over-striding (Robin et al., 2025a). Collectively, these adaptations reflect the human body’s compensatory strategies to achieve task completion while optimizing energy economy under mechanically adverse conditions.

Moreover, our study employed SPM1d for the continuous data analysis of time series. Traditional statistical methods simplify continuous data into the value of peak or average, potentially omitting critical waveform features (e.g., joint ROM, the process of ground reaction forces, and trajectory morphology) (Yona et al., 2024). The method revealed that significant differences in joint moments of the dominant leg predominantly occurred at the end of the stance phase (Figure 3), which is critical for generating speed. Our findings align with load-induced hip moment alterations on stair ascent during the stance phase (Zijun et al., 2024), suggesting a common compensatory mechanism. Therefore, this supports the concept that individuals with hip-dominant running under heavy loads amplify the strength of the thigh to counteract propulsion inefficiencies caused by restricted ROM in the lower limbs (Michelle et al., 2018).

In addition, vertical GRF characteristics are critical for understanding the biomechanical mechanisms of running, which also plays a key role in preventing musculoskeletal injuries and evaluating rehabilitation processes. Previous studies have shown that, unlike parachute/sled training (Sam et al., 2021), load carriage predominantly amplifies vertical COM oscillation. Usually, the vertical GRF is composed of the first peak when the heel is cushioned and the second peak when the toe is in propulsion. The peak vertical GRF increases significantly with the increase in running speed. As expected, peaks increase significantly with running speed and load magnitude (Lin et al., 2021; Bruno et al., 2018). Our study confirms this, indicating that the increase in peak impact force and propulsion force is proportional to the increase in load, which is consistent with previous studies (Tian et al., 2017). Our results also demonstrate that activation of the thigh muscles of the dominant leg is increased during loaded sprinting. This especially emphasizes the importance of the rectus femoris and biceps femoris (Table 2). Musculoskeletal disorders are the most common injury type in firefighters, often affecting the ankle, knee, and leg (Noah et al., 2022). Thus, to address these risks, a volume of evidence also suggests that improving load carriage performance requires a dual focus on aerobic fitness and strength training (Robin et al., 2025a).

## 5 Limitation

In this study, professional firefighters were selected to perform loaded sprinting under pre-fatigue and post-fatigue states to explore the biomechanical characteristics of this specialized movement. Strict standardization procedures and control of the preparation time before the experiment were used, while the fatigue protocol of the maximal graded exercise test may not uniformly induce comparable fatigue across diverse firefighter populations. Therefore, future studies should investigate sport-specific fatigue protocols that mimic the mixed metabolic demands of actual firefighting duties. Besides, the real environment of the actual task was not considered in the laboratory environment test. It is suggested that future research should further investigate the influence of factors such as environment, clothing, task type, duration, and symmetry on rescue effectiveness, possibly by employing machine learning algorithms for gait pattern recognition to provide practical solutions and further reveal the underlying mechanisms.

In recent years, machine learning algorithms have been used for pattern recognition and classification of gait features to emphasize the uniqueness of gait patterns.

## 6 Conclusion

When sprinting with external weight constraints, gait metrics are negatively impacted, and compensating kinetic strategies increase push-off power from the hip and ankle of the dominant leg. During loaded sprinting, these adaptations are further reflected in the firefighters’ increased activation of the muscles around the hip joint. Crucially, these adaptations occur independently of fatigue state. The primacy of propulsion mechanics emphasizes the demands for task-specific training targeting thigh and shank power transfer efficiency under load. It provides a mechanistic foundation for evidence-based training to enhance occupational performance and mitigate biomechanical risk factors.

## Data Availability

The raw data supporting the conclusions of this article will be made available by the authors, without undue reservation.
